# The Enhanced Photoluminescence Properties of Carbon Dots Derived from Glucose: The Effect of Natural Oxidation

**DOI:** 10.3390/nano14110970

**Published:** 2024-06-03

**Authors:** Pei Zhang, Yibo Zheng, Linjiao Ren, Shaojun Li, Ming Feng, Qingfang Zhang, Rubin Qi, Zirui Qin, Jitao Zhang, Liying Jiang

**Affiliations:** 1 Henan Key Lab of Information-Based Electrical Appliances, College of Electrical and Information Engineering, Zhengzhou University of Light Industry, Zhengzhou 450002, China; zhangpei@zzuli.edu.cn (P.Z.); zhengyb_2018@163.com (Y.Z.); shaojunli1997@163.com (S.L.); fengming9851@163.com (M.F.); qingfang@zzuli.edu.cn (Q.Z.); qirubin@zzuli.edu.cn (R.Q.); 2019005@zzuli.edu.cn (Z.Q.); zhangjitao@zzuli.edu.cn (J.Z.); 2 School of Electronics and Information, Academy for Quantum Science and Technology, Zhengzhou University of Light Industry, No. 136 Ke Xue Avenue, Zhengzhou 450002, China

**Keywords:** carbon dots, dual-fluorescent, natural oxidation, oxygen-containing groups, fluorescence mechanism

## Abstract

The investigation of the fluorescence mechanism of carbon dots (CDs) has attracted significant attention, particularly the role of the oxygen-containing groups. Dual-CDs exhibiting blue and green emissions are synthesized from glucose via a simple ultrasonic treatment, and the oxidation degree of the CDs is softly modified through a slow natural oxidation approach, which is in stark contrast to that aggressively altering CDs’ surface configurations through chemical oxidation methods. It is interesting to find that the intensity of the blue fluorescence gradually increases, eventually becoming the dominant emission after prolonging the oxidation periods, with the quantum yield (QY) of the CDs being enhanced from ~0.61% to ~4.26%. Combining the microstructure characterizations, optical measurements, and ultrafiltration experiments, we hypothesize that the blue emission could be ascribed to the surface states induced by the C–O and C=O groups, while the green luminescence may originate from the deep energy levels associated with the O–C=O groups. The distinct emission states and energy distributions could result in the blue and the green luminescence exhibiting distinct excitation and emission behaviors. Our findings could provide new insights into the fluorescence mechanism of CDs.

## 1. Introduction

As a new type of fluorescent nanomaterials, carbon dots (CDs) have garnered increasing attention over the past two decades due to their numerous advantages, including their multi-color emission, excellent biocompatibility, high quantum yield (QY) and wide luminescence range. These properties enable their extensive applications in various potential fields such as bioimaging [1], sensors [2,3], light-emitting diodes (LEDs) [4,5], luminescent solar concentrators [6,7] and lasers [8,9]. Although the photoluminescence (PL) properties of CD materials have been extensively explored, a unified fluorescence mechanism remains elusive. This is due to the multiple emission centers and diverse electronic structures induced by the different particle sizes, conjugation lengths, surfaces, passivation, etc. [10,11]. Notably, the role of oxygen-containing groups bonded to the CD surface in fluorescence emission is a significant factor, as the oxygen atoms are readily incorporated into the CD structure during the preparation process [10,11].

The effects of oxygen-containing groups on the luminescence efficiency and wave-length of CDs have been intensively explored [12,13,14,15,16,17,18,19]. Several studies have found that the QY of CDs can be substantially enhanced by removing oxygen-related groups through violent reduction reaction treatments [12,13,14]. However, contrary findings have also been reported by other researchers [15,16,17]. Additionally, the emission wavelength of CDs tends to redshift with an increase in oxygen-containing groups, as the optical bandgap of CDs narrows after incorporating these species [18,19]. During the oxidation or reduction process, the reagents used not only alter the carbon cores but also significantly modify the surface configurations of CDs, further complicating the luminescence mechanism.

Therefore, it is essential to comprehensively understand the effect of oxygen-related surface states on the luminescence mechanisms of CDs with multiple emissions, which is crucial for tailoring their surface chemistry. In this work, dual-fluorescent (blue and green) CDs are synthesized using a one-step ultrasonication method. The surface configurations of the CDs are gently modified through aerial oxidation, resulting in an enhancement of the QY from about 0.61% to 4.26% as the oxidation degree increases. Interestingly, the intensity of both blue and green emissions shows similar variation trends with the amounts of their respective oxygenous groups during the oxidation process. Based on microstructure characterizations, optical measurements, and ultrafiltration experiments, we hypothesize that the blue emission, which exhibits excitation-independent characteristics, can be attributed to C–O and C=O groups, while the green luminescence possessing the excitation-dependent feature is likely associated with O–C=O groups. Furthermore, the emission states induced by different oxygenous groups have distinct distribution and energy levels within the bandgap of small carbonaceous materials or CDs, leading to the blue and the green luminescence exhibiting different excitation-emission properties. The enhanced luminescence efficiency can be explained by two factors: the reduction in the number of oxygen-centered radicals, as evidenced by the electron paramagnetic resonance (EPR) spectra, and the increase in the density of oxygen-related emission states.

## 2. Materials and Methods

### 2.1. The Synthesis of Dual-Fluorescent CDs

All the regents used in this experiment were purchased from Sigma Aldrich (Shanghai, China), including glucose, NaOH (97%), ethanol, HCl (37%), and MgSO_4_ (97%). The CDs were synthesized through the following steps: (1) Mix the aqueous glucose solution (1 mol/L, 10 mL) with the NaOH solution (1.5 mol/L, 10 mL), then treat the mixture using the ultrasonication method for 4 h. The ultrasonic power and frequency were set at 400 W and 50 Hz, respectively. (2) After the ultrasonic treatment, add HCl to adjust the pH of the mixture to 7. (3) Gradually add 100 mL of ethanol using a piston burette while stirring. Subsequently, add about 17 g of MgSO_4_ (wt%) to the mixture. The stirring speed and time were set at 2000 r/min and 20 min, respectively. (4) Finally, obtain the CDs in ethanol/water by removing the precipitate [20].

**Figure 1:**
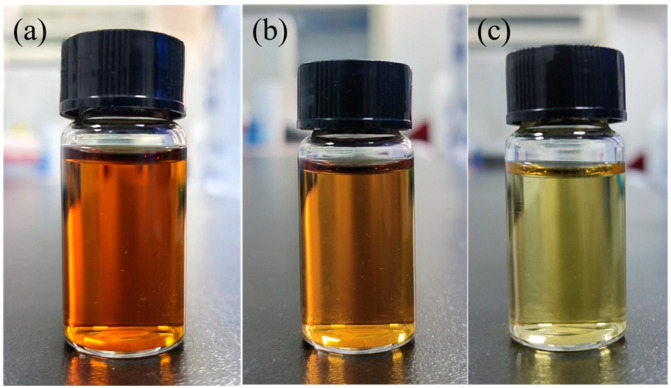
Photographs of (**a**) SA1, (**b**) SA2, and (**c**) SA3 under the irradiation of daylight.

After synthesizing the CDs, we transferred 60 mL of the CD solution into a 125 mL wide-mouth bottle and placed it in a low-temperature chamber. The sample was oxidized by the air in the bottle, and we termed this process as natural oxidation. It is important to note that the bottle was covered with a cap but not sealed. The same initial sample was subjected to different oxidation times and labeled as SA1 (as-synthesis), SA2 (1–2 months) and SA3 (3–6 months). The color of the CD solution clearly changed from dark brown to the light brown during the oxidation process, as displayed in Figure 1. It is essential to clarify that these three samples are not prepared in separate batches (except the samples used for the measurements of nuclear magnetic resonance); rather, the same initial sample underwent different oxidation periods, which were utilized for the microstructure characterizations and optical measurements. It is worth noting that even for the CDs from different batches, their luminescent characteristics will undergo the aforementioned three stages during the long period of oxidation, and the changing process is also repeatable.

### 2.2. Characterization and Optical Experiments

Transmission electron microscopy (TEM) images were obtained by utilizing a FEI Tecnai G2 F30 TEM (Thermo Fisher Scientific, Waltham, MA, USA) operating at a voltage of 300 kV. Fourier-transform infrared (FTIR) spectra were measured using a TENSOR II FTIR Spectrometer (Bruker, Ettlingen, Germany) with a spectral resolution of 4 cm^−1^ in the range of 4000 to 400 cm^−1^. X-ray photoelectron spectroscopy (XPS) analysis was performed on an Escalab 250Xi (Thermo Electron, Waltham, MA, USA) using Al K*α* (1486.6 eV) monochromatic radiation via the excitation source. Raman spectra ranging from 1000 to 1500 cm^−1^ were recorded via a LabRAM HR Evolution micro Raman system (HORIBA Scientific, Vénissieux, France) with a spectral resolution of 2 cm^−1^, utilizing a diode-pumped solid-state laser with the 532 nm line as the excitation source. X-band electron paramagnetic resonance (EPR) spectra were acquired via a Bruker EMX-plus spectrometer (Bruker, Germany) operating at 9.4 GHz under the center field of 3250 G. The ^1^H and ^13^C nuclear magnetic resonance (NMR) spectra were collected using a Bruker AM X400 (Bruker, Germany) spectrometer operating at a frequency of 400 and 100 MHz, respectively.

The UV-vis absorbance spectra ranging from 220 to 600 nm were obtained using a Shimazu UV-3600 spectrophotometer (Shimadzu, Kyoto, Japan). The steady photoluminescence (PL) spectra were measured using an F-7000 (Hitachi, Tokyo, Japan) fluorescence spectrometer with a 450 W Xenon lamp as the excitation source. Both the excitation and emission slits were set to 2.5 nm, and the photomultiplier tube (PMT) was utilized to detect the emission light. Time-resolved photoluminescence (TRPL) spectra were recorded with an FLS980 fluorescence spectrophotometer (Edinburgh, UK). The equipped excitation lasers were an EP-LED-360 picosecond (ps) pulse LED (*λ*_exc_ = 360 nm and pulse duration ~950 ps) and an EPL-450 ps pulse diode laser (*λ*_exc_ = 405 nm and pulse duration ~75 ps), respectively. The absolute fluorescence QY was measured using an FLS980 fluorescence spectrophotometer with an integrating sphere.

## 3. Results

### 3.1. Characterizations of the CDs

**Figure 2:**
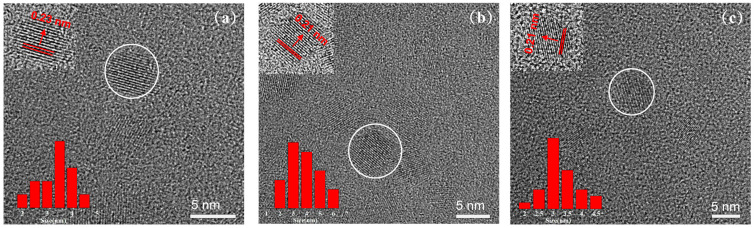
TEM images of the (**a**) SA1, (**b**) SA2, and (**c**) SA3 samples, and the upper and lower left insets are the HRTEM images (the circled CDs) and size distribution of the CDs, respectively.

Figure 2 shows the TEM images of SA1, SA2, and SA3 with different oxidation degrees. The high-resolution TEM (HR-TEM) images of the CDs display the well-resolved lattice fringes, and the corresponding interplane spacing for the three samples is about 0.21 nm (upper left insets), which is close to the value of the (100) plane of graphitic carbon [7,8]. The particle size distributions of the three samples range from 2 to 6 nm, and the CDs exhibit a quasi-spherical shape with an average dimeter of ~3 nm. The CDs retain good solubility in water even after a long period of oxidation, as displayed in the Supplementary Materials (Figure S1). However, due to the relatively wide size distribution of the dots, it is challenging to discern the variations in their size and shape during the long oxidation time.

**Figure 3:**
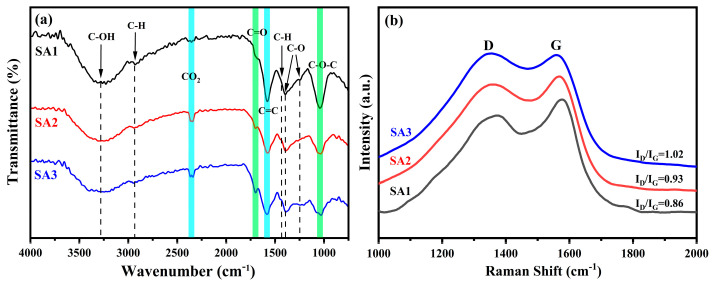
(**a**) FTIR and (**b**) Raman spectra of SA1 (black curve), SA2 (red curve), and SA3 (blue curve).

The chemical bonding and surface configurations of the CDs can be derived from FTIR and Raman spectra measurements, as shown in Figure 3. In the FTIR spectra (Figure 3a), three main absorption peaks at approximately 1045, 1394, and 1579 cm^−1^ are observed, corresponding to the C–O–C [21], C–O [22], and C=C functional groups [21], respectively. In addition, a broad absorption band centered around 3286 cm^−1^ indicates the presence of the stretching vibrations of C–OH bonding [23]. Several small peaks at around 1243, 1448, 1700, and 2942 cm^−1^ arise from the vibrational absorption of C–O [18], C–H(CH_2_) [24], C=O/COOH [25,26], and C–H (CH_3_) [24] groups, respectively. The presence of the numerous hydrophilic groups endows the CDs with good solubility in water.

To get the FTIR signal, the liquid CDs were freeze-dried into powders. After a long period of oxidation, the C=O (1700 cm^−1^) signal for SA3 becomes slightly more pronounced due to the weakened absorption of the C=C bonds. However, its absorption intensity has not changed significantly. Additionally, the absorption intensities of C–O–C and C–O decrease from the SA1 to the SA3 sample, which may arise from the different concentrations of CDs used when measured, which will be further discussed in the UV-absorption section. Additionally, a new absorption peak at approximately 2347 cm^−1^, associated with CO_2_, appears in the SA2 and SA3 samples. This may result from CO_2_ adsorption or the high oxidation degree on the surface of the CDs [27].

Figure 3b shows the Raman spectra of SA1, SA2, and SA3. Two peaks are observed around 1373 cm^−1^ (D-band) and 1578 cm^−1^ (G-band). The G-band is associated with the graphitic sp^2^ carbons, whereas the D-band is related to the sp^3^ disorder carbons [15,27]. Notably, the intensity ratio *I*_D_/*I*_G_ increases from 0.86 to 1.02 with longer oxidation time, indicating that more oxygen atoms incorporate into the CD structures and form oxygen-containing groups [15,27].

**Figure 4:**
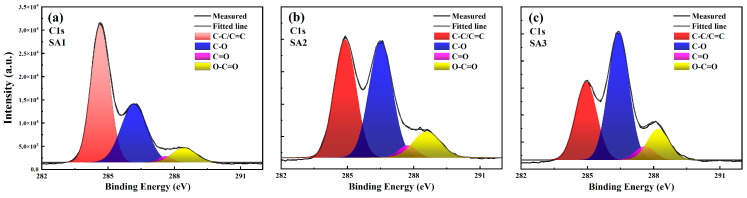
The C 1s high-resolution XPS spectra of (**a**) SA1, (**b**) SA2, and (**c**) SA3.

To elucidate the evolution of the elemental composition and chemical groups of CDs during different oxidation stages, XPS measurements were carried out. Figure 4 displays the C 1s and O 1s XPS spectra of the SA1, SA2, and SA3 samples, from which the percentage of C and O atoms could be calculated. The fractions of O (C) content in SA1, SA2, and SA3 are 30.95% (69.05%), 39.88% (60.12%), and 42.40% (57.60%), respectively. This demonstrates that the as-synthesized CDs (SA1) already contain a considerable number of oxygen atoms, and the oxygen content in the CDs increases gradually when the natural oxidation time is prolonged, indicating that many more O atoms participated in the formation of chemical groups at the surface of the CDs.

The high-resolution C 1s XPS spectra of the three samples can be deconvoluted into four Gaussian components: C=C/C–C (graphitic carbon) at ~284.8 eV [28], C–O at ~286.2 eV (epoxy) [15], C=O at ~287.6 eV(carbonyl) [29], and O–C=O at ~288.5 eV (carboxyl) [30,31]. The percentages of graphitic carbon and oxygenated carbon are listed in Table 1. As the oxidation time increases, there is a gradual decline in the proportion of graphitic carbon from 48.9% to 30.4%, while the content of oxygenated carbon (C–O, C=O and O–C=O) increases. The progressively increasing presence of the C=O groups is consistent with the FTIR analysis. However, no obvious change is observed in the O–C=O groups between the SA2 and SA3 samples. The high-resolution O 1s XPS spectra of the SA1 and SA2 samples consist of two fitting peaks at around 532.7 eV and 531.4 eV (Figure S2), which are attributed to the C=O and C–OH/C–O–C groups [31], respectively. Their ratios are listed in Table S1. Moreover, for the SA3 sample, in addition to the C=O and C–OH/C–O–C groups, a new band centered at 536 eV appears, which could be attributed to the absorption of H_2_O or O_2_ [32,33].

The ^1^H and ^13^C NMR spectra of CDs in DMSO further confirm the presence of abundant by-products during the synthetic process, as shown in Figure S3. In the ^1^H NMR spectra (Figure S3a), two main regions can be observed: one in the range of 1–2.5 ppm, corresponding to the sp^3^ C–H protons, and the other between 3 and 6 ppm, originating from the protons attached to the hydroxyl, ether, and carbonyl groups [34]. In addition, the peak at 8.44 ppm could be attributed to the formate [35]. In the ^13^C NMR spectra (Figure S3b), four main regions are identified: peaks between 20 and 80 ppm originating from aliphatic (sp^3^) carbon atoms [34], signals from 80 to 85 ppm attributed to the carbons linked with ether groups [34], peaks in the range of 90–102 ppm arising from the anomeric carbons of *α*-pyranoses [36], and signals between 165 and 180 ppm ascribed to the carboxyl and carbonyl groups [34,37]. Additionally, the peak near 105 ppm is due to the anomeric carbons in *β*-pyranoses [36].

The analysis of the ^1^H and ^13^C NMR spectra reveals that in addition to the presence of hydroxyl, ether, and carbonyl groups, by-products such as formate and pyranoses are also detected. This suggests that the synthesized CD solution contains numerous organic compounds. A multitude of by-products are generated during the synthesis process of CDs, which causes their NMR signals to cluster together, as depicted in Figure S3. For the NMR measurements, the samples were dealt with spin-drying to substantially reduce the effects of water on the results. In addition, the concentration of small carbonaceous materials gradually decreases during the oxidation process. Consequently, even if the microstructure of the sample changes during the oxidation process, distinguishing these changes remains challenging due to the inability to control the concentration of CDs and small carbonaceous materials when measured.

### 3.2. Steady Optical Properties

**Figure 5:**
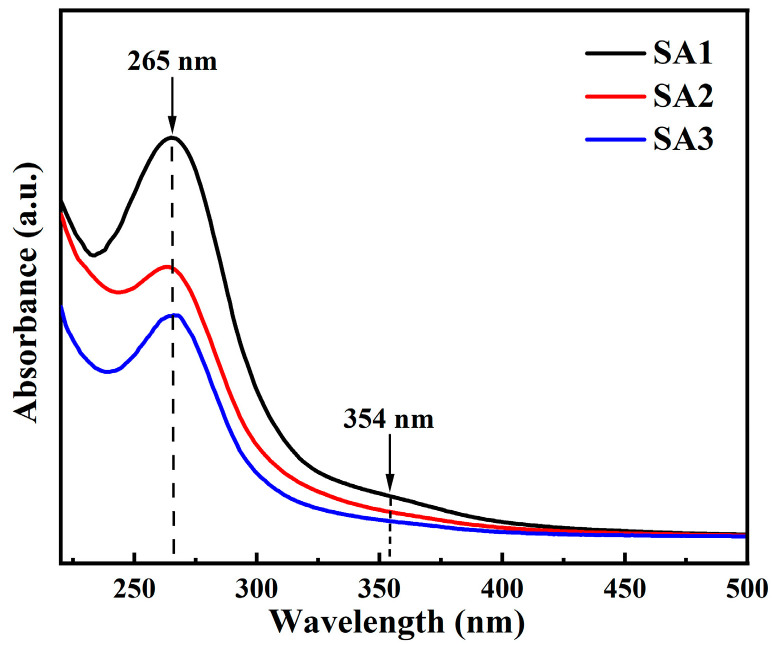
The UV-vis absorbance spectra of SA1 (black curve), SA2 (red curve), and SA3 (blue curve).

Figure 5 displays the UV-Vis absorbance spectra of the three samples, the main absorption peak at 265 nm can be attributed to the π–π* transitions of C=C, while the shoulder band centered at approximately 354 nm stems from the n–π* transitions of C=O [20,31,38]. It is evident that the overall absorbance intensity significantly decreases with the increasing oxidation time.

According to the Raman and XPS analyses, as the oxidation degree of the CDs increases, the content of the graphitic carbon decreases, accompanied by an increase in the fraction of oxygenated carbon. Although this could explain the weakening of π–π* transitions in the graphitic carbon (C=C), it is difficult to interpret the reduced absorption intensity associated with the C=O groups, which seemingly contradicts the increment of the carbonyl and carboxyl groups. During the synthesis of the CDs, we consider that a significant amount of small carbonaceous materials (<1 nm) were generated when the glucose underwent condensation, dehydration, polymerization, or carbonization processes [10,11]. Parts of these small carbonaceous materials (including aromatic rings, small carbon clusters, and CDs with smaller dot size), with oxygen-containing groups might be transformed into a CO_2_ structure after long-time oxidation. This transformation could lead to a decrease in CD concentration, which may be the main reason for the decrease in the overall absorption intensity as well as the reduction in the amount of the C–O–C and C–O contents observed in the FTIR measurements. In addition, the increasing transparency of the CD solution can further support this hypothesis.

**Figure 6:**
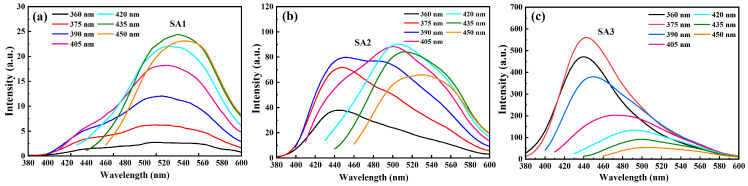
The PL spectra of (**a**) SA1, (**b**) SA2, and (**c**) SA3 under different excitation wavelengths.

It is well established that the PL properties of the CDs can be significantly modified by changing the oxidation degree of the CDs’ surface structure, leading to variation in the electronic structure or emission states. Figure 6 illustrates the PL spectra of SA1, SA2, and SA3 under different excitation wavelengths, it is interesting to observe that the evolution of the PL spectra of CDs during the oxidation process can be evidently divided into three stages. For the as-synthesized sample (SA1), the PL intensity of the green emission (~505 nm) is stronger than that of the blue luminescence (~435 nm), as depicted in Figure 6a and Figure S4a. In this excitation region, the peaks of the two emission bands change very little, while their intensity gradually increases. The optimal excitation wavelengths for the two emission peaks are about 392 and 422 nm, respectively, which could be acquired in the PL excitation (PLE) spectra as depicted in Figure S5a.

Compared with the SA1 sample, the intensity of the blue emission band in the SA2 is stronger than that of the green luminescence under 360 nm excitation, as illustrated in Figure 6b. The PL intensity of both two emission bands increases within the excitation range between 360 and 390 nm, but the intensity of the green luminescence becomes comparable to that of the blue emission when excited by 390 nm. An obvious red-shift in the green luminescence is observed when the excitation wavelength is further increased.

The SA3 sample exhibits clear excitation-dependent behavior, the PL peak red-shifts with the increasing excitation wavelength. Notably, the blue emission dominates within the excitation range of 360–390 nm, reaching a maximum PL intensity under ~365 nm excitation. In addition, both blue and green emissions in SA3 exhibit nearly identical optimal excitation wavelengths (~365 nm), as depicted in Figure S5c. Conversely, the optimal excitation wavelengths for the blue and the green luminescence between the SA1 and SA2 samples differ significantly, as shown Figure S5.

**Figure 7:**
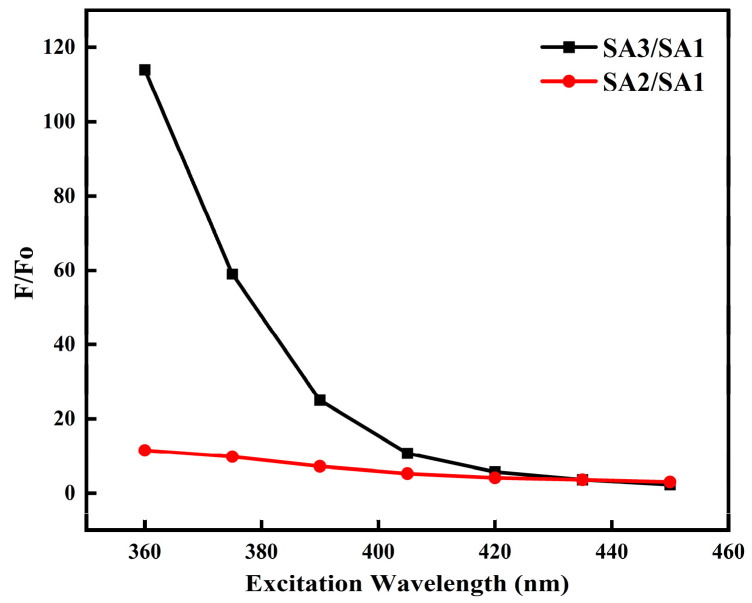
The ratio of the PL intensity of SA2/SA1 (red circle) and SA3/SA1 (black square) within the excitation range of 360–450 nm.

More importantly, the PL intensity of the CDs remarkably enhances in the blue region after a long oxidation period, as displayed in Figure 7. However, after being increase several times initially, the intensity of the green emission remains relatively stable, even when the oxidation time is prolonged further. This demonstration underscores the distinct dominant emission states among the three samples due to their varied excitation and emission properties.

There are lots investigations about the PL mechanisms of dual-fluorescent CDs; several excitation and recombination models have been proposed to explain the luminescence processes, including the size effect (usually referred to as the sp^2^ nanodomain), surface states, molecule states, the synergistic effect, etc. [10,11,39,40,41,42]. A large number of results demonstrate that the emission states induced by the oxygen-containing groups play a significant role in the optical properties of CDs [12,13,14,15,16,17,18,19,39,40,41,42]. However, these oxygenous functional groups have diverse structural compositions, including the hydroxyl (–OH), carbonyl (C=O), carboxyl (–COOH), and epoxy (C–O–C) groups. Due to the complex surface configurations, the origins of luminescence in CDs remain controversial, particularly for the blue and green emissions.

Wen et al. found that the fluorescence of CDs consisted of two distinct emission bands. One was associated with the intrinsic band located in the blue region, which was ascribed to the sp^2^ sub domains. The other originated from the surface states introduced by abundant oxygen-containing functional groups, emitting longer-wavelength lights [43]. Wang’s group suggested that the hydroxyl group decorated at the surface of graphene quantum dots (GQDs) played an important role in the blue fluorescence (430 nm), while the synergy effect of the hydroxyl group and the adjacent carbonyl group might cause the green emission (530 nm) [44]. Li and co-workers proposed that the blue emission centered at 440 nm was related to the -OH-related surface hybridized states, whereas the green or yellow-green was associated with the C–O–C (epoxy) groups [15].

In addition, numerous groups have hypothesized that oxygen-containing functional groups, acting as the non-radiative recombination centers, would suppress the intrinsic state emission associated with the sp^2^ sub domains, leading to the low QY [12,13,14]. In contrast, several studies have suggested that the QY of CDs could be enhanced after an oxidation reaction [15,16,17,45]. For instance, Li’s group reported that the PLQY of GQDs could be enhanced through oxidation treatment using H_2_O_2_ and UV light irradiation, with the emission color shifting from green to yellow-green, attributed to an increase in C–O–C (epoxy) groups [15]. Han et al. reported that the fluorescence QY of GQDs was increased through a post-oxidation method employing H_2_O_2_, resulting in a red-shift of the PL peak from 450 nm to 510 nm, ascribed to the increase in carbonyl and carboxyl groups [16]. Dong and co-workers found that the PL intensity of graphene oxide quantum dots (GOQDs) significantly decreased as the reduction degree increased, due to the decrease of the oxygen-containing groups, particularly the -COOH and -OH groups [45].

In our case, the broad PL band excited by the 375 nm could also be decomposed into a blue emission component and a green luminescence band, as depicted in Figure S4. Additionally, the QY of CDs increases from ~0.61% to ~4.26% after a long period of oxidation. The intensity of the blue emission is over one hundred times stronger than that of the as-synthesis sample, with the green luminescence also showing several-fold increases. According to the density functional theory (DFT) calculations conducted by Eda’s group, the sp^2^ cluster consisting of about five fused aromatic rings is responsible for emitting the blue light [46]. Sk et al. suggested that the emission wavelength could be tuned from ~400 to 572 nm by varying the dimeter of GQDs from 0.92 to 1.39 nm [47]. However, the TEM images reveal that after long-time oxidation, the particle sizes of the CDs still show a broad distribution ranging from 2 to 6 nm, with an average diameter of about 3 nm (>100 aromatic rings). Within these size ranges, the PL of CDs is predominantly in the red region. We speculate that the blue and green luminescence may originate from the CDs with smaller dot sizes or small carbonaceous materials.

**Figure 8:**
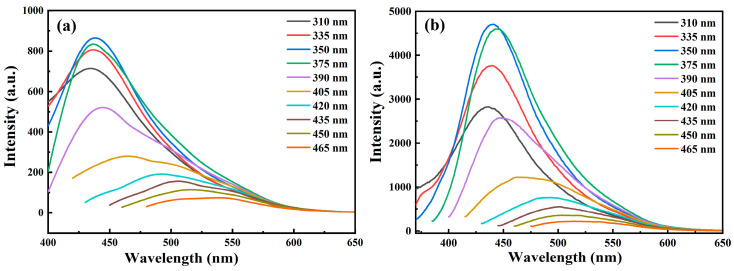
The PL spectra of the SA2 experienced relatively (**a**) short and (**b**) long oxidation times after ultrafiltration treatment when excited by different wavelengths. Both the samples are in the second oxidation stage.

To testify this hypothesis, we use the ultrafiltration treatment with a Millipore (3 kDa, cutoff) to filter the particles larger than 2 nm. The SA2 sample that underwent relatively short and long oxidation times were utilized, and their PL spectra after ultrafiltration treatment are displayed in Figure 8. Interestingly, the PL spectra of filtered SA2 samples at both oxidation stages exhibit similar excitation-dependent behaviors to those observed in the SA3 sample. Within the excitation range of 310–375 nm, there is a broad emission band spanning from 400 to 600 nm. Gaussian fitting allowed for the segmentation of this spectrum into two emission bands: a blue band centered at approximately 435 nm and a green band peaking around 490 nm, as depicted in Figure S4. The peak of the blue band remains almost unchanged under excitation wavelengths from 310 to 375 nm. Upon further increasing the excitation wavelength, the green luminescence peak gradually shifts towards longer wavelengths (red-shift). We also compare the PL spectra of the residual samples by the Millipore filter with those of the original solution (Figure S6), and find that the spectral features of the residue do not significantly differ from those of the original solution. Based on these ultrafiltration results, we consider that the blue emission primarily originates from small carbonaceous materials.

Moreover, the change in the surface configurations and particle sizes of the CDs tailored through different oxidation methods (chemical or natural) may impart distinct optical properties to the CDs [14,15,16,17]. Xu’s group used an HNO_3_ reflexing method to oxidize the CDs and noted that the oxidation not only increased the oxygen content but also led to the formation of an oxygen-containing loose shell on the surface of the CDs [14]. Li et al. observed an increase in the amount of C-O-C bonds after they oxidized the graphene quantum dots (GQDs) with the hydrogen peroxide (H_2_O_2_) and UV light; notably, a high concentration of H_2_O_2_ could accelerate the oxidation process, potentially damaging or breaking down the conjugated structure of GQDs [15]. Additionally, Han and co-workers synthesized oxidized GQDs using a post-oxidation treatment with H_2_O_2_, which led to an increase in C=O and COOH groups while the proportion of C–O groups (epoxy and hydroxyl) decreased [16]. Srivastava’s group altered the oxygen degree of the CDs via aerial oxidation, resulting in an increased density of hydroxyl groups on the CD surface and the transformation of C=N-H bonds into C=N bonds, with no significant changes to morphology [17].

In our study, we observed a gradual decline in the ratio of C–C/C=C, accompanied by an increase in the proportion of C–O and C=O groups, with the fraction of O–C=O groups reaching its maximum after an initial increase. We hypothesize that as the oxidation degree increases, the content of C–C/C=C components gradually diminishes, potentially leading to a slight reduction in the size of the small carbonaceous materials (<1 nm).

### 3.3. Time-Resolved PL (TRPL) Optical Properties

**Figure 9:**
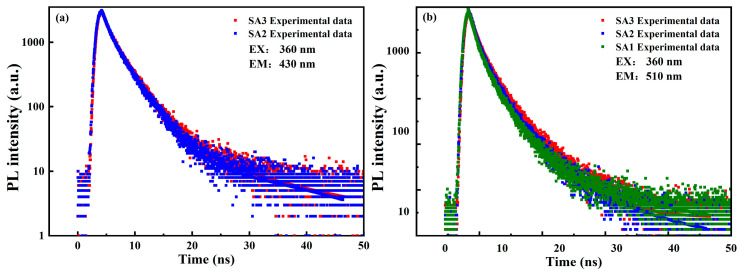
For time-resolved PL spectra of SA1, SA2, and SA3 with 360 nm as the excitation wavelength, the detected emission wavelengths are (**a**) 430 and (**b**) 510 nm, respectively.

To further explore the luminescence process, measurements of the time-resolved PL (TRPL) spectra of CDs are carried out. Figure 9 displays the PL decay curves of three samples under ps LED light (*λ*_exc_ = 360 nm and pulse duration ~950 ps) excitation, the monitoring emission wavelengths are 430 and 510 nm, respectively. The PL decay curves could be well fitted by a bi-exponential function, typically used to evaluate the lifetime of CDs [48].

(1) It=A1exp−tτ1+A2exp−tτ2

where *A*_1_ and *A*_2_ represent the amplitude of two decay components, respectively. The *τ*_1_ and *τ*_2_ are the lifetimes of two decay components, respectively. The average decay lifetime $\bar{\tau }$ can be calculated by the followed equation [48]:

(2) $$\bar{\tau }=\frac{A_{1}\tau_{1}^{2}+A_{2}\tau_{2}^{2}}{A_{1}\tau_{1}+A_{2}\tau_{2}}$$

The fitted and calculated values from Equations (1) and (2) are listed in Table 2. Analysis of the PL decay curves and data in Table 2 reveals a slight increase in the lifetime of CDs after a longer period of oxidation, with a decrease in the proportion of the shorter lifetime and an increase in the longer decay process. The PL dynamics of sample SA1 could not be detected due to the weak blue emission signal under the excitation of the ps LED pump source. For a comprehensive comparison of the recombination dynamics of the blue emission, the TRPL spectra of the three samples excited by a ps pulse diode laser (*λ*_exc_ = 405 nm and pulse duration ~75 ps) are also measured, as depicted in Figure S7. The detected emission wavelengths are 470 and 530 nm, respectively. The characteristics of the average lifetime and the proportions of the two decay processes are similar to those excited at 360 nm, except for the average lifetime of SA3 detected at 530 nm, which shows a difference (see Table S2).

Although several groups have also identified two distinct lifetimes in CDs, the origins of these decay processes are still a subject of debate [49,50,51]. Liu et al. attributed the fast component (~1.32 ns) to the recombination process involving the intrinsic states, and ascribed the slow lifetime (~7.89 ns) to the emissions from defect states [49]. However, Zhao and co-workers proposed that the faster component (1.29–1.73 ns) was associated with the radiative recombination of the eigenstates, while the slower lifetimes (2.51–7.16 ns) arising from the non-radiative process were related to the surface defects [50]. In addition, Byun’s group suggested that the two lifetimes originated from the nonradiative recombination (1.17–3.15 ns) and radiative recombination processes (5.7–8.52 ns) of oxygen-induced defects, respectively [51].

### 3.4. Surface Defects and Luminescence Mechanisms of CDs

**Figure 10:**
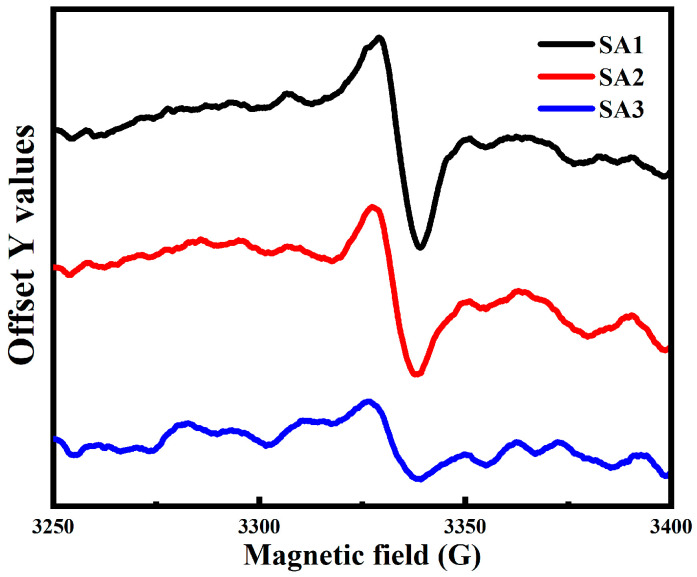
The EPR spectra of SA1 (black curve), SA2 (red curve), and SA3 (blue curve).

In order to deeply investigate the relationship between the microstructure and PL properties of CDs at different oxidation stages, the EPR spectra of the three samples are measured. The trend in the concentration of the unpaired electrons or free radicals is illustrated in Figure 10. For each measurement, about 5 mg of freeze-dried powders are used. It is evident that the intensity of the EPR signal decreases gradually from SA1 to SA3, indicating a reduction in the concentration of unpaired electrons or free radicals with an increasing oxidation time. The *g*-value, determined from the EPR spectrum, is approximately 2.005, typically associated with the oxygen-centered radicals [52,53]. Additionally, the EPR spectral linewidth is about 1 mT, suggesting that the free radicals predominantly arise from oxygen-containing groups connected to the sp^3^ hybridization of carbon [54].

## 4. Discussion

To uncover the impact of oxygen on the luminescence mechanisms of CDs, several key issues need clarification. Firstly, after a long period of oxidation, the blue emission components of CDs become dominant under high-energy photon irradiation. Secondly, the blue emission displays an excitation-independent characteristic, whereas the green luminescence exhibits excitation-dependent behavior. Thirdly, the CD solution (SA2) obtained through ultrafiltration treatment shows PL characteristics similar to those of the sample with a higher oxidation degree.

First of all, according to the UV-vis absorbance and PLE spectra of CDs, the absorption peaks centered at 265 and 354 nm scarcely change. However, the optimal excitation wavelengths for the blue and green emissions undergo a blue shift after a long period of oxidation, eventually converging around 365 nm. This wavelength, where the maximum luminescence intensity is achieved, overlaps with the n–π* transitions of C=O. The close proximity of the optimal excitation wavelengths to the absorption peak may be a crucial factor in enhancing the PL intensity of the CDs. Additionally, both the blue and the green luminescence have two decay processes: fast and slow components. Notably, the PL dynamics of the detected blue and green fluorescence show minimal differences, suggesting that the luminescence centers possess similar recombination processes (lifetimes). Despite this, the QY of CDs with higher oxygen contents remains low compared to those with high-quality surface passivation. This observation leads to the hypothesis that the fast component is likely due to non-radiative recombination behaviors, while the slow component may result from the radiative recombination processes of photon-generated carriers.

Moreover, the blue emission was thought to arise from the small carbonaceous materials, as indicated by discussions of the PL properties of CDs after ultrafiltration treatment. The XPS results reveal that the proportions of C–O and C=O groups gradually increase when the oxidation time is prolonged, while the ratio of the O–C=O group reaches its maximum after an initial rise. It is noteworthy that the PL intensity of the blue emission exhibits a similar variation trend to the amounts of the C–O and C=O groups. Furthermore, the changing trend in the intensity of the green emission accords well with the variation in the O–C=O contents. Therefore, combining these analytical findings, we propose that the blue emission is associated with the C–O and C=O groups decorating the edges of small carbonaceous materials. Since the green luminescence has similar PL dynamics to the blue fluorescence, it is likely attributed to the O–C=O groups.

Righetto et al. synthesized three types of CDs (pCDs, oCDs, and mCDs) through solvothermal pyrolysis using ortho-, meta-, and para-phenylenediamine, respectively. The resulting CD solutions were carefully purified and subsequently characterized using fluorescence correlation spectroscopy and time-resolved electron paramagnetic resonance techniques. The findings revealed that the emission properties were predominantly dominated by free fluorescent molecular by-products [55]. By analyzing the ^1^H and ^13^C NMR spectra of CDs in DMSO, we find that the synthesized CD solution contains various organic compounds. These molecular products could impact the optical properties of CDs.

Secondly, the blue emission displays excitation-independent behavior, whereas the green luminescence is excitation-dependent. This suggests significant differences in the emission states and energy distributions within the bandgap of the carbon dots (CDs), induced by their respective oxygen-containing groups. Sun’s group also observed similar phenomena and noted that CDs with different oxygen contents exhibit distinct excitation-dependent behaviors [56]. These observations were attributed to the competition between various transition processes arising from various oxygenous groups. It is speculated that the carboxyl as well as carbonyl functional groups are related to the green waveband, while the blue emission is possibly associated with the surface functional groups in the planes or at the edges of the CDs, such as the hydroxyl groups. In our case, the blue emission dominates the fluorescence when the carbonaceous materials have a smaller particle size or a higher oxidation degree, indicating that the small carbonaceous materials are more prone to oxidation due to their larger surface-to-volume ratio during the natural oxidation process. A substantial portion of carbon is transformed into the C–O and C=O groups, increasing the density of relevant emission states and significantly enhancing the blue emission intensity. The increase in the proportion of oxygen-related states at high energy levels may cause the blue shift in the optimal excitation wavelengths. Furthermore, the relatively uniform distribution of the C–O and C=O groups on the surface of small carbonaceous materials may contribute to the excitation-independent characteristics of the blue emission. Conversely, the intensity of the green emission is generally stronger in CDs with larger particle sizes or lower oxidation degrees. The non-uniform coverage of O–C=O groups might result in a wide distribution of the related energy levels, leading to excitation-dependent behavior.

Additionally, both concentration and aggregation are important factors affecting the optical properties of CDs. To illustrate this, the CD solutions are diluted 1-, 2-, 3-, 4-, and 5-fold with water, and the corresponding PL spectra are depicted in Figure S8. The sample exhibits a PL peak centered at approximately 440 nm under 360 nm of excitation, which is almost unchanged regardless of the dilution fold. The PL intensity reaches its maximum when the solution is diluted 3-fold, then decreases as the dilution fold increases further. Since the blue emission primarily stems from the oxygen-containing functional groups (C–O and C=O) on the surface of the small carbonaceous materials, the significant increase in PL intensity upon dilution highlights the presence of the aggregation within these materials. A broad PL band peaked at around 500 nm is obtained when the sample is excited at 420 nm. The PL peak experiences a slight blue-shift when the dilution fold is two, but remains almost unchanged with further dilution. The PL intensity decreases gradually as the dilution fold increases, yet this decrease is not directly proportional to the dilution fold. We attribute the green luminescence primarily to the oxygenous groups (O–C=O) decorated at the surface of the CDs with larger particle sizes or lower oxidation degrees. Unlike the small carbonaceous materials, the aggregation phenomenon is less pronounced in CDs with larger particle sizes. Furthermore, the concentration of small carbonaceous materials decreases with the increase in the oxidation degree, which also contributes to a rise in PL intensity.

Finally, particle size plays a crucial role in influencing the PL properties of CDs. With the increasing oxidation degree, the surface of the CDs undergoes further oxidation, accompanying a reduction in CD size. This change can alter the coupling degree between the π-electron system and the oxygen-related surface states, leading to the blue shift in the PL peak. As illustrated in Figure 6, no blue shift is observed in the blue emission, likely due to the relatively broad size distribution of the CDs. It remains challenging to identify the effects of particle size variation on the PL peak shift. We implemented a straightforward ultrafiltration treatment using a Millipore (3 kDa, cutoff) to filter the particles larger than 2 nm. The PL spectra after ultrafiltration treatment are shown in Figure 8. When excited at 360 nm, the blue emission dominated the PL spectra, the spectral characteristics of the filtered sample differ markedly from those of the unfiltered sample. Nevertheless, the PL peak of the blue band remains nearly unchanged, indicating that even small carbonaceous materials possess a certain degree of particle size distribution.

For the residual samples in the ultrafiltration tube, their spectral characteristics remained identical to those of the original CD solution. One possible explanation for this is that the small carbonaceous materials are not entirely filtered out, with some still physically adhering to the surfaces of the CDs with larger dot sizes [39]. Another possibility is that the sp^3−^- and sp^2−^-hybridized carbons on the CD surface, decorated with the oxygen functional groups, may also emit blue and green light when excited.

**Figure 11:**
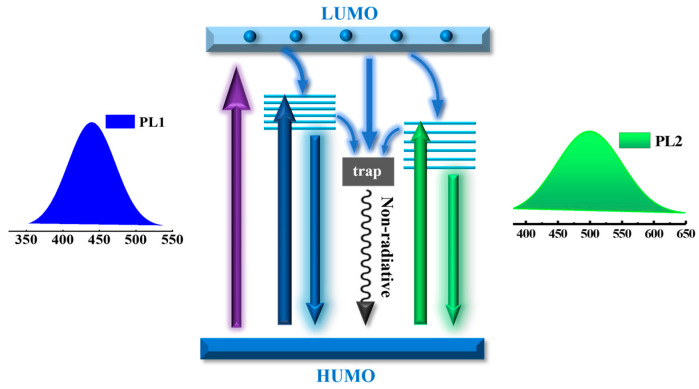
A schematic diagram of the PL mechanisms of the oxidized-CDs.

Moreover, there still exists competition among different emission states and non-radiative traps, as illustrated in the schematic model of the excitation and recombination process in Figure 11. Initially, the photo-excited electrons are generated by transitioning from the n-state orbitals (HUMO) to the π* orbitals (LUMO) of C=O by absorbing the high-energy photons. These electrons quickly relax into oxygen-related emission states or get trapped by the non-radiative defects. The green and the blue luminescence stems from the radiative recombination of the electron-hole pairs at corresponding oxygen-related energy levels. The EPR spectra confirm that the concentration of oxygen-centered radicals would decrease after a long period of oxidation, potentially reducing the non-radiative traps and enhancing the QY of CDs. However, only a slight increase in the average lifetime is observed in samples with higher oxygen contents. We speculate that the increase in oxygenous groups may enhance the density of oxygen-related emission states and improve the relaxation rate between energy levels. This could counterbalance the effect of reduced non-radiative traps on the recombination process, resulting in only a small change in the lifetime of the CDs.

## 5. Conclusions

The dual-fluorescent (blue and green emission) CDs are prepared using a simple ultrasound method. The surface configuration of the CDs could be softly altered through natural oxidation. The QY of the CDs increases significantly from approximately 0.61% to 4.26% after a long period of oxidation. Based on microstructural characterizations, optical measurements, and ultrafiltration experiments, we consider that the C–O and C=O groups are prone to form at the surface of small carbonaceous materials during the natural oxidation process, leading to the enhancement of the blue emission. Conversely, the green emission, associated with O–C=O groups, is stronger in CDs with larger particle sizes or lower oxidation degrees. The emission states induced by their corresponding oxygenous groups display distinct distribution and energy levels within the bandgap of small carbonaceous materials or CDs, leading to excitation-independent blue emission and excitation-dependent green luminescence. Furthermore, the increase in emission states caused by corresponding oxygen-containing groups as well as the reduction of the amount of oxygen-centered radicals contribute to the enhanced QYs of the CDs. This work provides an in-depth understanding of the role of oxygen in the optical properties of CDs.

## Figures and Tables

**Table 1:** The XPS data analyses of the C 1s spectra.

	C–C/C=C	C–O	C=O	O–C=O
SA1	48.9%	33.2%	4.8%	13.1%
SA2	37.2%	39.2%	7.3%	16.3%
SA3	30.4%	44.2%	9.3%	16.1%

**Table 2:** The fitting parameters and average lifetimes of the three samples under the excitation *λ*_exc_ = 360 nm.

Emission	430 nm			510 nm		
Sample	SA1	SA2	SA3	SA1	SA2	SA3
*τ*_1_ (ns)	-	1.41	1.23	1.50	1.54	1.21
*A*_1_ (%)	-	61.47	51.33	79.81	75.99	58.96
*τ*_2_ (ns)	-	3.73	3.64	5.04	5.00	4.34
*A*_2_ (%)	-	38.53	48.67	20.19	24.01	41.04
$\bar{\tau }$	-	2.90	3.01	3.12	3.29	3.44

## Data Availability

The data presented in this work are available upon request via email to the corresponding author.

## Supplementary Materials

The following supporting information can be downloaded at: https://www.mdpi.com/article/10.3390/nano14110970/s1, Figure S1: TEM images of (a) SA1, (b) SA2, and (c) SA3 (50 nm scale bar); Figure S2: O1s high-resolution XPS spectra of (a) SA1, (b) SA2, and (c) SA3; Figure S3: (a) ^1^H NMR and (b) ^13^C NMR spectra of SA1 (black line), SA2 (red line), and SA3 (blue line); Figure S4: The Gaussian fitting results of PL spectra excited by 375 nm for (a) SA1, (b) SA2, and (c) SA3; Figure S5: PLE spectra of (a) SA1, (b) SA2, and (c) SA3. The emission wavelengths are 435 (black line) and 505 nm (red line), respectively; Figure S6: The PL spectra of the residual samples for SA2 experienced relatively (a) short and (b) long oxidation times; The PL spectra of the SA2 experienced (c) short and (d) relative long oxidation times before ultrafiltration treatment; Figure S7: Time-resolved PL spectra of SA1, SA2, and SA3 with the 405 nm as the excitation wavelength, the emission wavelengths are (a) 470 and (b) 530 nm, respectively; Figure S8: The PL spectra of SA2 at various dilution levels (1, 2, 3, 4 and 5-fold) under (a) 360 nm and (b) 420 nm of excitation; (c) The PL intensity of SA2 under 360 nm (black square) and 420 nm (red circle) of excitation as a function of the dilution fold; Table S1: XPS data analyses of the O 1s spectra; Table S2: The fitting parameters and average lifetimes of the three samples under the excitation *λ*_exc_ = 405 nm.
