# Supplementary material for: The Enhanced Photoluminescence Properties of Carbon Dots Derived from Glucose: The Effect of Natural Oxidation
Source: Nanomaterials (Basel). 2024 Jun 3;14(11):970. doi: 10.3390/nano14110970 (PMC11174097; doi:10.3390/nano14110970)
Supplement: Supplementary file 1 [file nanomaterials-14-00970-s001.zip › nanomaterials-2987330-supplementary.pdf]

Supplementary Materials

The Enhanced Photoluminescence Properties of Carbon Dots Derived from Glucose: The Effect of Natural Oxidation

Pei Zhang [1], Yibo Zheng[1], Linjiao Ren [1,*], Shaojun Li [1], Ming Feng [1], Qingfang Zhang [1], Rubin Qi [1], Zirui Qin [1], Jitao Zhang [1] and Liying Jiang [2*]

1 Henan Key Lab of Information-based Electrical Appliances, College of Electrical and Information Engineering, Zhengzhou University of Light Industry, Zhengzhou 450002, China; zhangpei@zzuli.edu.cn (P.Z.); zhengyb_2018@163.com (Y.Z.); shaojunli1997@163.com (S.L.); fengming9851@163.com (M.F.); qingfang@zzuli.edu.cn (Q.Z.); qirubin@zzuli.edu.cn (R.Q.); 2019005@zzuli.edu.cn (Z.Q.); zhangjitao@zzuli.edu.cn (J.Z.)

2 School of Electronics and Information, Academy for Quantum Science and Technology, Zhengzhou University of Light Industry, Zhengzhou 450002, China

* Correspondence: renlinjiao@zzuli.edu.cn (L.R.); jiangliying@zzuli.edu.cn (L.J.)

Electronic Supporting Information

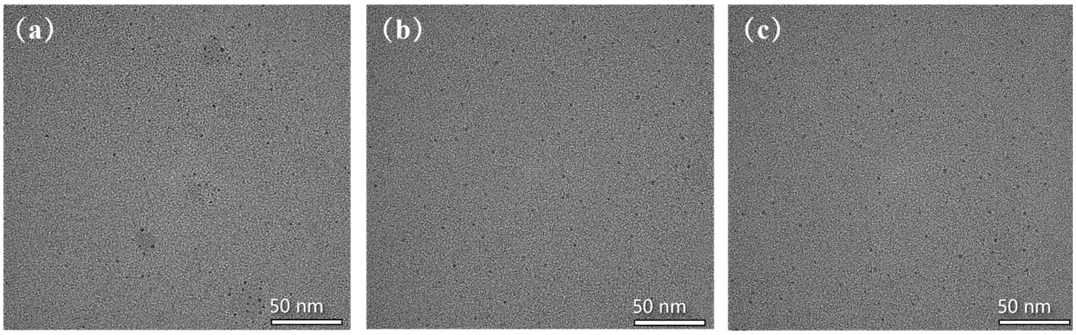

Figure S1. TEM images of (a) SA1, (b) SA2, and (c) SA3 (50 nm scale bar).

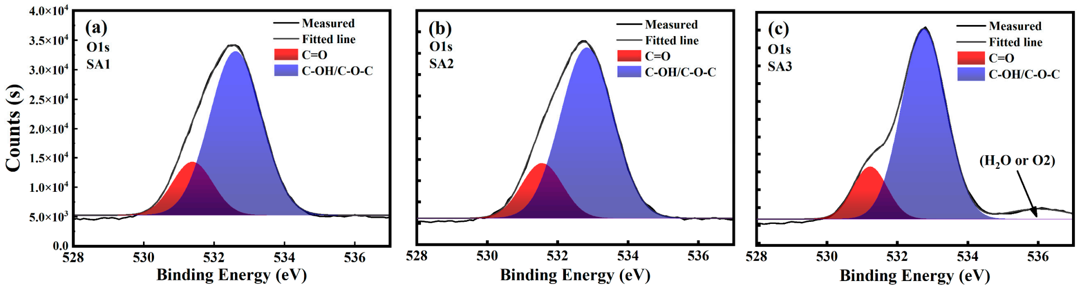

Figure S2. O 1s high-resolution XPS spectra of (a) SA1, (b) SA2, and (c) SA3.

Table S1. XPS data analyses of the O 1s spectra.

Sample	C=O	C-OH/C-O-C
SA1	20.3%	79.7%
SA2	20.0%	80.0%
SA3	17.4%	82.6%

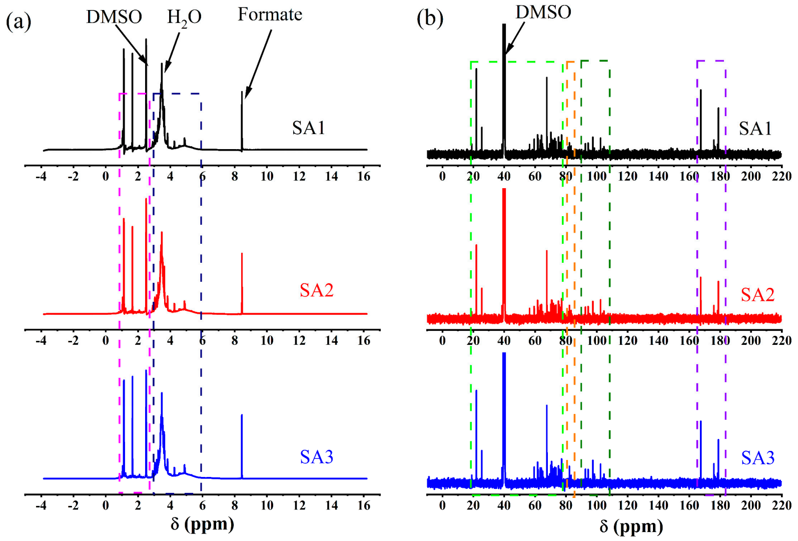

Figure S3. (a) ^{1}H NMR and (b) ^{13}C NMR spectra of SA1 (black line), SA2 (red line), and SA3 (blue line).

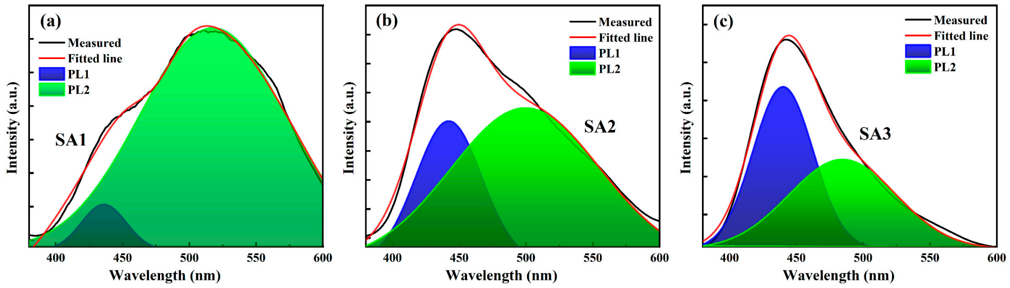

Figure S4. The Gaussian fitting results of PL spectra excited by 375 nm for (a) SA1, (b) SA2, and (c) SA3.

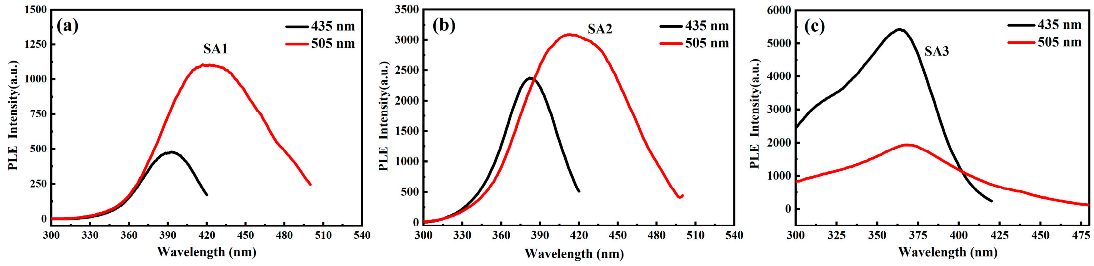

Figure S5. PLE spectra of (a) SA1, (b) SA2, and (c) SA3. The emission wavelengths are 435 (black line) and 505 nm (red line), respectively.

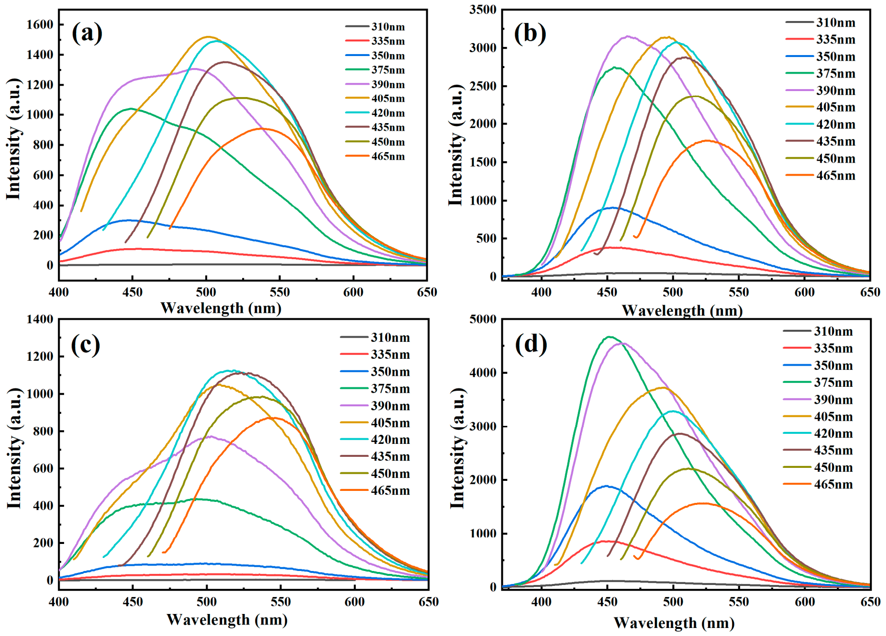

Figure S6. The PL spectra of the residual samples for SA2 experienced (a) short and (b) relative long oxidation times; The PL spectra of the SA2 experienced relatively (c) short and (d) long oxidation times before ultrafiltration treatment.

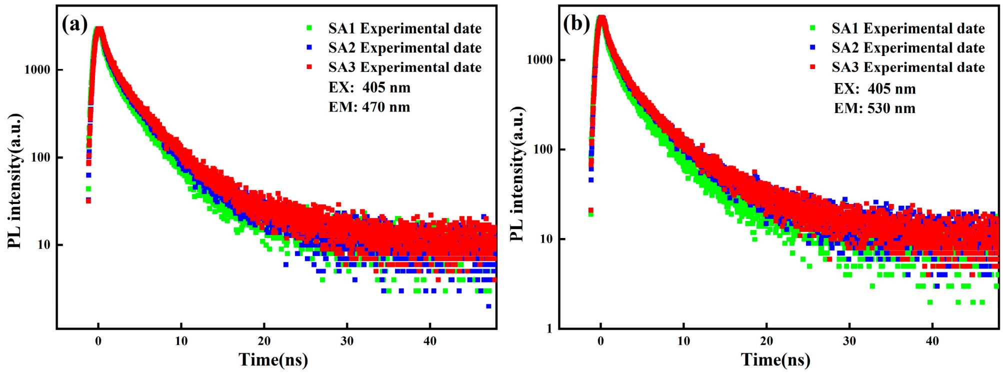

Figure S7. Time-resolved PL spectra of SA1, SA2 and SA3 with the 405 nm as the excitation wavelength, the detected emission wavelengths are (a) 470 and (b) 530 nm, respectively.

Table S2. The fitting parameters and average lifetimes of the three samples under the excitation λ_{exc} = 405 nm.

Emission	470 nm			530 nm		
Sample	SA1	SA2	SA3	SA1	SA2	SA3
τ_1 (ns)	0.82	0.66	0.79	0.91	1.04	0.92
A_1 (%)	65.55	54.71	56.48	74.41	70.12	65.08
τ_2 (ns)	3.61	3.50	3.73	4.09	4.49	4.23
A_2 (%)	34.45	45.29	43.52	25.59	29.88	34.92
$\bar{\tau}$	2.77	2.97	3.01	2.84	3.28	3.28

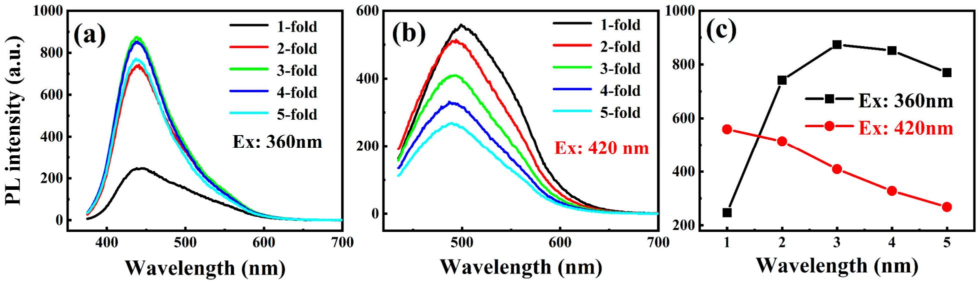

Figure S8. The PL spectra of SA2 at various dilution levels (1, 2, 3, 4 and 5-fold) under (a) 360 nm and (b) of 420 nm excitation; (c) The PL intensity of SA2 under 360 nm (black square) and 420 nm (red circle) of excitation as a function of the dilution fold.
